# Assessing the scientific quality of radiotherapy popularization videos on BiliBili and TikTok: a cross-sectional study

**DOI:** 10.3389/fpubh.2026.1668189

**Published:** 2026-02-13

**Authors:** Jingru Han, Xiao Li, Man Li, Xuanxuan Wu

**Affiliations:** 1 Department of Neurosurgery, Children’s Hospital of Chongqing Medical University, National Clinical Research Center for Child Health and Disorders, Ministry of Education Key Laboratory of Child Development and Disorders, Chongqing Key Laboratory of Pediatrics, Chongqing, China; 2 Research Center of Cancer Diagnosis and Therapy, Department of Oncology, The First Affiliated Hospital of Jinan University, Guangzhou, China

**Keywords:** radiotherapy, short video platforms, health communication, information quality, content reliability

## Abstract

**Background:**

With the proliferation of short video platforms such as BiliBili and TikTok, public reliance on these platforms for medical information has increased substantially. However, the absence of standardized content regulation raises serious concerns about misinformation, oversimplification, and variable quality in health communication. Radiotherapy, a cornerstone of cancer treatment alongside surgery and chemotherapy, is particularly vulnerable to such information quality issues due to its technical complexity and limited public understanding. This necessitates systematic evaluation of the scientific accuracy and reliability of radiotherapy content on these platforms.

**Methods:**

In this cross-sectional study, the top 100 Chinese-language videos related to “放射治疗” (radiotherapy) were collected from BiliBili and TikTok (total *n* = 200). Video quality and reliability were assessed via the Global Quality Score (GQS) and modified DISCERN tools. Nonparametric tests and Spearman correlation analyses were applied. Two independent radiotherapy specialists evaluated the content, with a third resolving discrepancy.

**Results:**

Overall video quality and reliability were moderate (median GQS = 3; DISCERN = 3). BiliBili demonstrated higher DISCERN scores (*p* < 0.05), reflecting superior reliability, whereas TikTok had marginally higher GQS scores. The BiliBili videos were significantly longer (median: 1,391.5 s vs. 98 s) and featured more systematic content, whereas the TikTok videos presented greater engagement (e.g., likes, shares, collects, comments). A positive correlation between video duration and DISCERN score was observed for BiliBili (*R* = 0.47, *p* < 0.0001), whereas TikTok showed a similar trend for GQS (*R* = 0.49, *p* < 0.0001). There was a lack of significant associations between interaction metrics and quality scores.

**Conclusion:**

This study evaluated 200 radiotherapy videos on BiliBili and TikTok. BiliBili showed higher reliability (DISCERN), whereas TikTok excelled in terms of user engagement. Recommendations include optimizing scientific communication, platform quality-based algorithms prioritizing authoritative content, and enhancing public media literacy. The findings can guide improvements in digital medical education.

## 1 Introduction

Radiotherapy, one of the three core approaches to cancer treatment (alongside surgery and chemotherapy), occupies an essential position in clinical application. Its core principle is to use high-energy ionizing radiation (e.g., X-rays, *γ*-rays, protons, or electron beams) to directly damage the DNA structure of cancer cells or to generate reactive oxygen radicals through ionization, which indirectly leads to DNA double-stranded breaks, thus inhibiting the proliferative ability of tumor cells and inducing their apoptosis (1). Modern radiotherapy employs high-energy ionizing radiation to target cancer cells while minimizing damage to surrounding healthy tissue through precise image guidance and dose optimization techniques (2, 3). Applications range from curative treatment of early-stage cancers to palliative care for metastatic disease (4, 5). Although technological advancements have markedly reduced the side effects of radiotherapy, acute reactions (e.g., radiation dermatitis, mucositis) and late complications (e.g., radiation pneumonitis, intestinal fibrosis) still need to be prevented and controlled by multidisciplinary management strategies, including evidence-based guideline-based skin care (e.g., barrier restorative use), nutritional support, glucocorticoid application, and targeted rehabilitation training (6, 7). Moreover, despite significant technological advances that have improved safety and efficacy, radiotherapy continues to be portrayed negatively in media and public discourse, often emphasizing historical side effects while overlooking modern innovations (8). This negative portrayal may contribute to treatment hesitancy and suboptimal decision-making among patients.

In the digital health era, short video platforms such as BiliBili and TikTok have become primary sources of medical information for the public in China (9). These platforms have lowered the threshold of understanding medical knowledge through fragmented content, but they also carry risks owing to the lack of professional review mechanisms. On the one hand, some videos contain misinformation (e.g., exaggerating the efficacy of radiotherapy, ignoring contraindications or side effects), oversimplification (e.g., simplifying complex radiotherapy techniques into “painless cures”), and even commercial misinformation (promoting unproven alternative therapies; promotion of untested alternative therapies); on the other hand, the diverse backgrounds of content creators (including physicians, self-publishing bloggers, and laypersons), with notable differences in their scientific validity and authority, may mislead patients’ decision-making and delay standardized treatment (10). Previous studies have assessed the quality of popular science videos on TikTok and BiliBili for a variety of diseases, such as liver cancer, thyroid nodules, and inflammatory bowel disease, and the results revealed that the quality of information on such platforms varies, with average credibility scores generally lower than those of professional medical resources (11–13). Given radiotherapy’s technical complexity and the existing negative media bias (8), understanding how this treatment is represented on popular video platforms is essential for developing effective public health communication strategies and protecting patients from harmful misinformation.

Therefore, this study focused on two mainstream platforms, BiliBili and TikTok, and screened a total of 200 short videos on radiotherapy science. To systematically assess video quality and reliability, this study employed two internationally validated instruments. The Global Quality Score (GQS) is a 5-point scale that evaluates overall video quality based on information flow, comprehensiveness, and usefulness for patients (14). The DISCERN tool, originally developed for written health information, assesses reliability through criteria including clarity of information sources, treatment benefit/risk balance, and evidence of bias (15). These complementary tools allow comprehensive evaluation of both content quality and clinical decision-support value.

## 2 Materials and methods

### 2.1 Data collection

**Figure 1:**
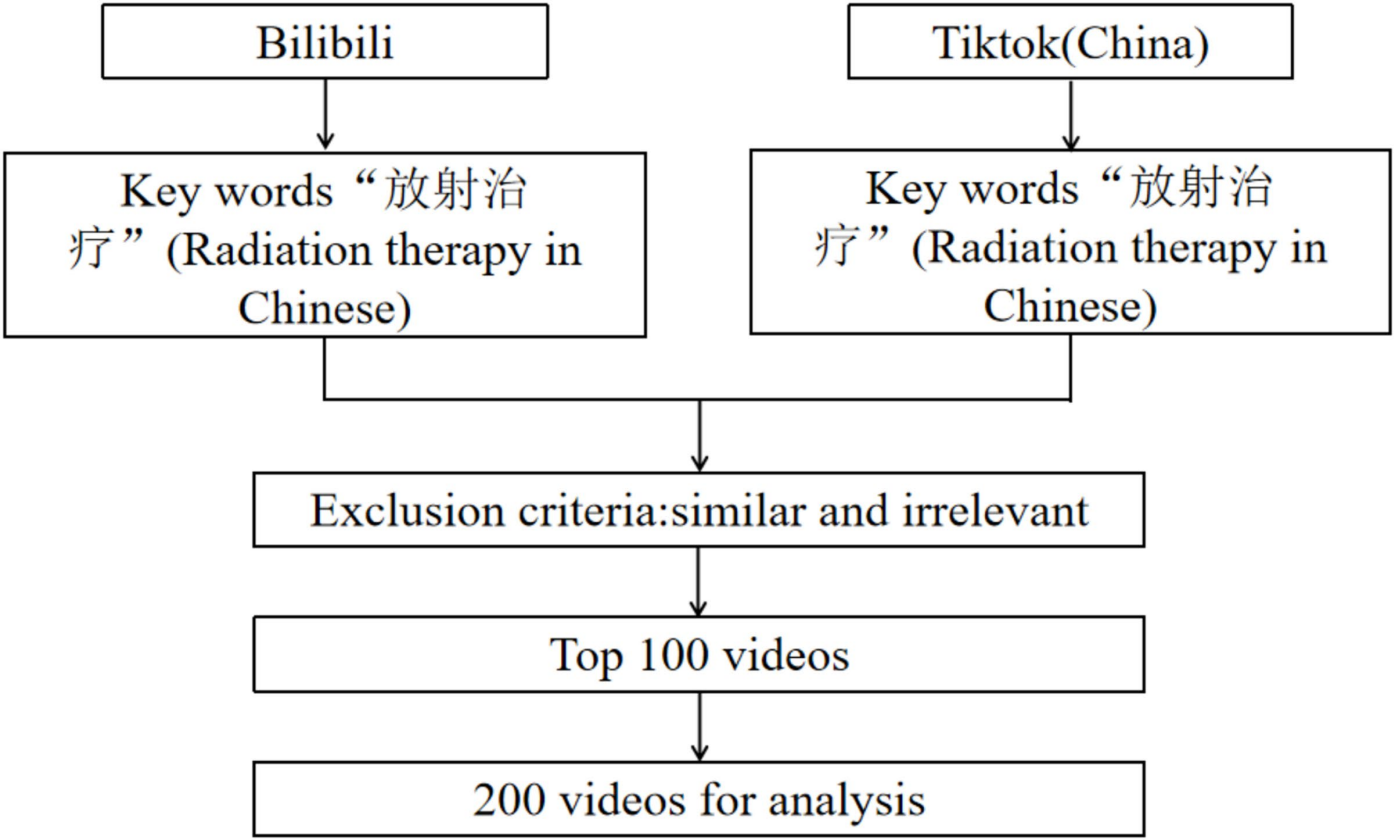
Flowchart of this research.

For each platform, we collected the first 100 videos appearing in search results when using the keyword ‘放射治疗’ (radiotherapy), sorted by the platform’s default ranking algorithm (which prioritizes relevance and engagement metrics). Videos were accessed using newly registered accounts without viewing history to minimize personalization bias (Figure 1). The researchers extracted basic information from these videos, including the source, content and format, duration (in seconds), number of likes, favorites, shares, comments, and time of posting.

### 2.2 Ethical statement

This study only involves the Chinese version of TikTok (Douyin) data. The relevant materials have been fully read and compliance with the《“抖音”用户服务协议》(https://www.douyin.com/draft/douyin_agreement/douyin_agreement_user.html?ug_source=sem_baidu&id=6773906068725565448) has been ensured. The research plan complies with all the regulations stipulated in the “TikTok Research Tools Terms of Service” regarding data access, usage, anonymization, protection of minors, and publication review. The research team has read and understood these terms, and will strictly abide by them throughout the entire research process. In the research, measures for data anonymization and de-identification have been implemented. No personal identity information of users is collected or stored.

### 2.3 Related methods and definitions

The global quality score (GQS) and modified DISCERN tool were used to assess the quality and reliability of the videos. The GQS is a globalized tool widely used for assessing the quality of video health information and is scored on a scale of five levels of poor quality, generally poor quality, moderate quality, good quality, and excellent quality, with a total score of 5. For specific details, see Supplementary Table S1 for categorization information. The modified DISCERN tool was used for video reliability assessment. When reviewing the videos, the researcher assessed whether the videos met the following criteria: clarity, relevance, traceability, robustness, and fairness. The above questions were answered with a score of yes (1 point) or no (0 points), and a cumulative score (0–5 points) was calculated as described in Supplementary Table S2. These two assessment methods are widely used in video-based platforms and disease information, so we chose this method for the assessment study in this study (14, 15).

To ensure the accuracy of the assessment of video quality and reliability, two independent radiotherapy specialists conducted the scoring assessment in this study. Moreover, for the objectivity and accuracy of the evaluation, the two radiotherapy specialists studied and understood the joint replacement guidelines again before the evaluation and discussed and harmonized the divergent details in the GQS and modified Discern tools. In cases of disagreement between the two radiotherapy specialists on the scoring of the same video, a third independent radiotherapy specialist viewed the video again, scored it, and used it for subsequent analysis.

### 2.4 Statistical analysis

Categorical variables are expressed as frequencies and percentages and were subjected to the chi-square test or Fisher’s exact test as needed. Owing to the random nature of the basic video information, the video data collected in this study exhibited a nonlinear distribution; thus, in this study, we used the median and interquartile range (IQR) to represent these data. For comparisons between two groups of nonnormally distributed data, the Mann–Whitney test was used; for comparisons between three or more groups, the Kruskal–Wallis H test was used. We used Cohen’s *κ* to quantify the agreement between two raters. *κ* values: *κ* > 0.8 indicates excellent agreement; 0.6 < *κ* ≤ 0.8 suggests good agreement; 0.4 < *κ* ≤ 0.6 indicates moderate agreement; and *κ* ≤ 0.4 indicates poor agreement. Moreover, because the data in this paper are nonnormally distributed, all the data included in the correlation analysis were analyzed via the Spearman model. SPSS 26.0 statistical software was used for all the statistical analyses, and a two-sided *p*-value < 0.05 was considered statistically significant.

## 3 Results

### 3.1 Videos characteristics

On the basis of the aforementioned inclusion and exclusion criteria, we collected 100 videos from each of the two short video platforms, TikTok and BiliBili, and the data were collected and analyzed by two independent radiotherapy specialists. We found that the likes, favorites, shares, and comments of videos on the TikTok platform were significantly greater than those on BiliBili, whereas in terms of video duration, videos on the BiliBili platform were longer than those on TikTok (Table 1).

**Table 1:** Video features on TikTok and BiliBili.

Variables	Total (*n* = 200)	TikTok (*n* = 100)	Bilibili (*n* = 100)	*p*-value
Like, M (Q₁, Q₃)	48.00 (7.00, 374.75)	353.00 (121.50, 887.50)	7.50 (2.00, 18.00)	<0.0001
Collect, M (Q₁, Q₃)	43.00 (8.00, 160.75)	102.00 (26.50, 491.25)	15.50 (4.00, 55.25)	<0.0001
Share, M (Q₁, Q₃)	15.00 (3.00, 104.25)	81.00 (11.50, 316.00)	5.50 (1.00, 18.00)	<0.0001
Comment, M (Q₁, Q₃)	4.00 (0.00, 33.25)	29.00 (11.75, 87.75)	0.00 (0.00, 1.00)	<0.0001
Time, M (Q₁, Q₃)	196.50 (88.75, 1371.25)	98.00 (61.75, 137.25)	1391.50 (414.25, 2540.00)	<0.0001

**Figure 2:**
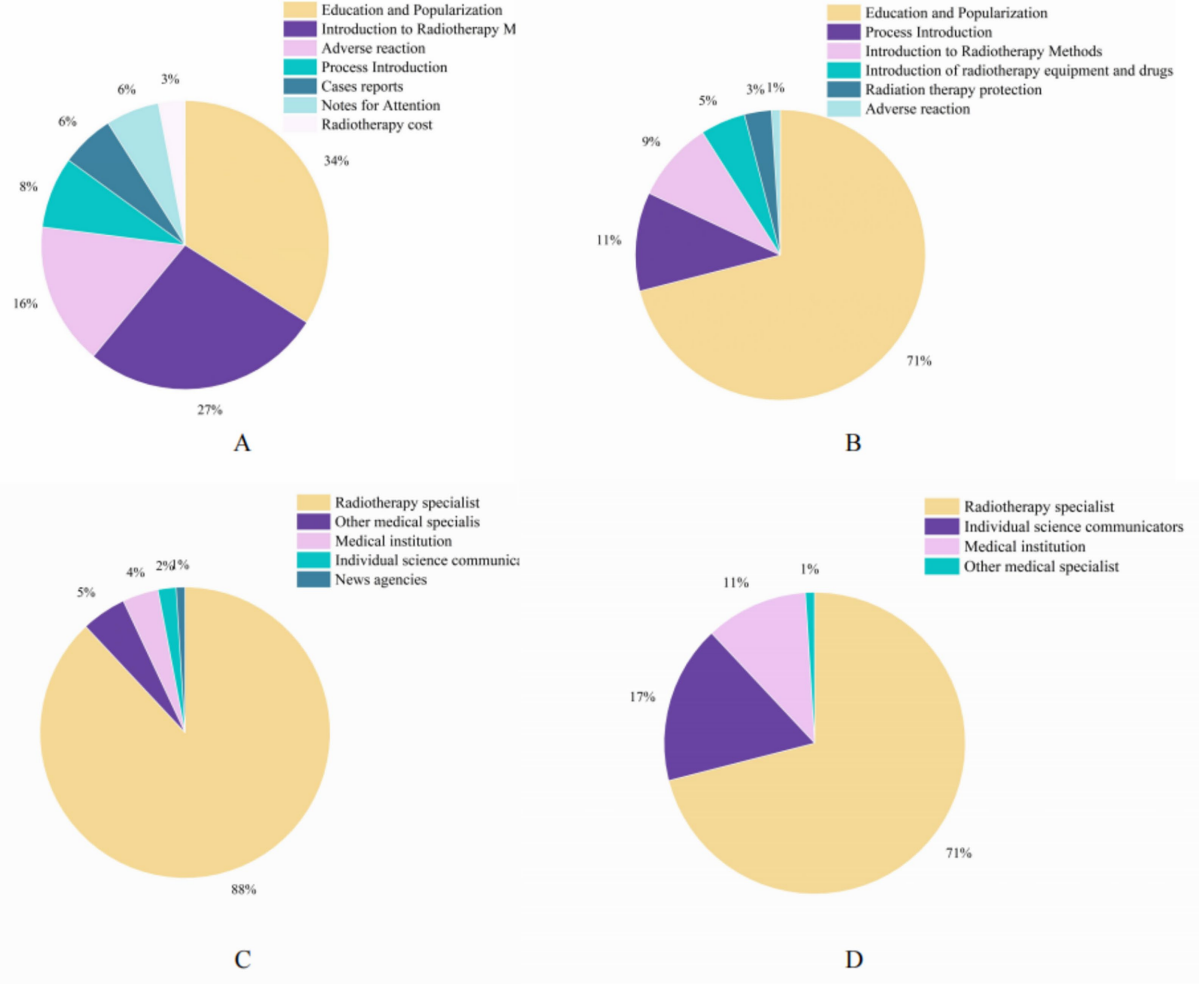
Percentage of videos based on video source, video content and video format on TikTok and BiliBili: **(A)** video content on TikTok, **(B)** video content on BiliBili, **(C)** video sources on TikTok, and **(D)** video sources on BiliBili.

In terms of video content, the education and popularization categories accounted for the largest share on both platforms, with TikTok accounting for 34%, whereas BiliBili accounted for 71% (Figures 2A,B). In terms of video sources, both platforms are dominated by videos uploaded by radiation oncologists (medical doctors specializing in radiotherapy), with TikTok accounting for 88% and BiliBili accounting for 81%. This category includes board-certified radiation oncologists; medical radiation technologists and radiotherapy technicians were categorized separately under ‘healthcare professionals’ if they explicitly identified their roles. Interestingly, 17% of BiliBili platform videos come from individual science communicators, far more than the 2% on the TikTok platform, which may indicate that the BiliBili platform has more individual science communicators (Figures 2C,D).

**Figure 3:**
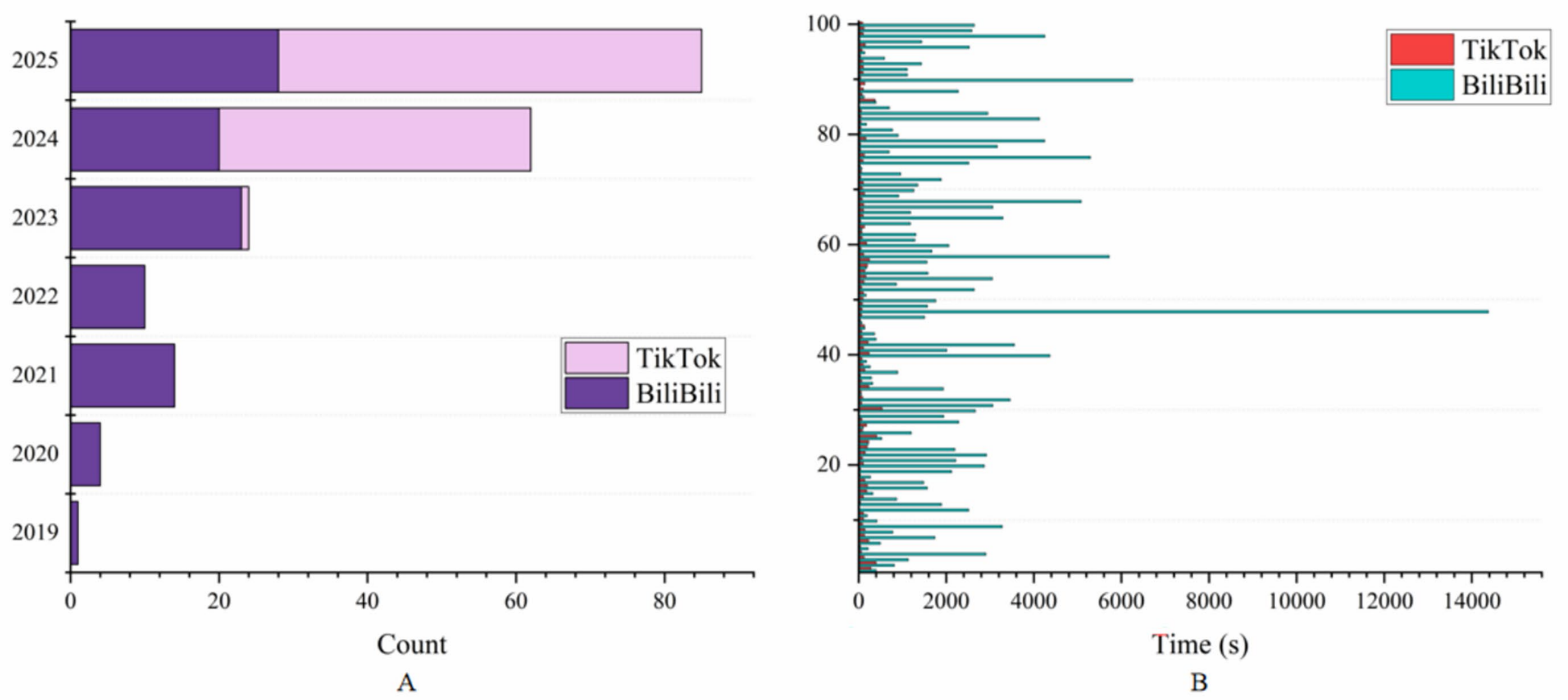
Distribution of video uploads according to year **(A)** and duration **(B)** on TikTok and BiliBili.

A comparison of the two platforms reveals that TikTok tends to have the highest values in terms of likes, collects, shares, and comments, whereas BiliBili is more homogeneous and TikTok is significantly greater than BiliBili (Supplementary Figure 1). Detailed video details on TikTok and BiliBili can be found in (Supplementary Tables S3, S4). In terms of video release time, videos on TikTok are significantly more time sensitive (Figure 3A). As shown in Figure 3B, in terms of video duration, the BiliBili video duration is much greater than that of TikTok.

### 3.2 Video quality and reliability assessment

**Figure 4:**
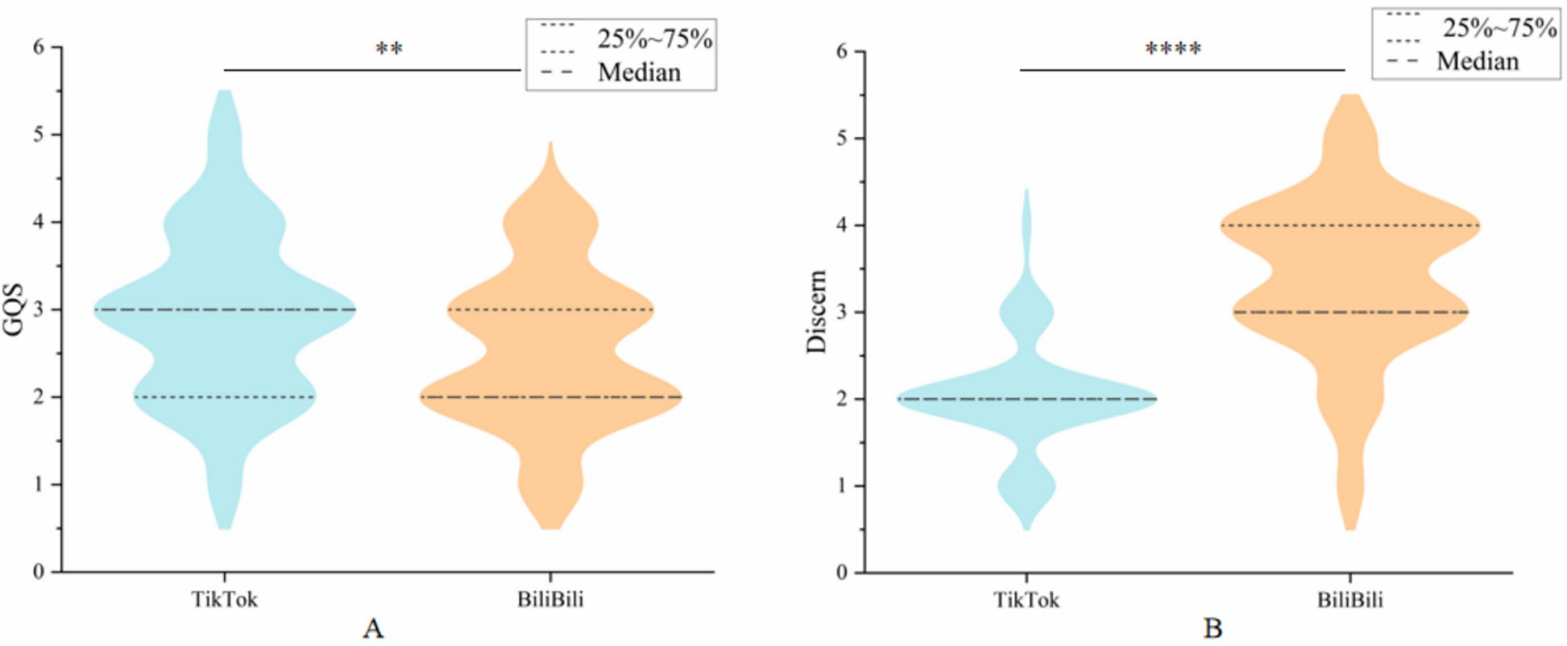
Comparison of videos from different platforms. **(A)** GQS score; **(B)** Modified DISCERN score.

Video quality was assessed via the GQS, and reliability was assessed via modified DISCERN tool scores. The Cohen kappa values for the GQS and modified DISCERN scores were 0.76 and 0.81, respectively, with good agreement. TikTok videos are of medium quality and poor reliability, in contrast to the BiliBili platform, which has poor video quality and medium reliability, with a median (IQR) GQS of 3 (2–3) for TikTok and 2 (2–3) for BiliBili. The median (IQR) concern scores were 2 (2–2) for TikTok and 3 (3–4) for BiliBili. Interestingly, TikTok is significantly higher than the BiliBili platform in terms of quality, whereas for the modified Discern scores, the BiliBili scores are significantly higher than the TikTok scores, and both are significantly different from each other. In conclusion, both platforms have their own advantages in terms of video quality and reliability (Figure 4).

### 3.3 Spearman’s correlation analysis

**Figure 5:**
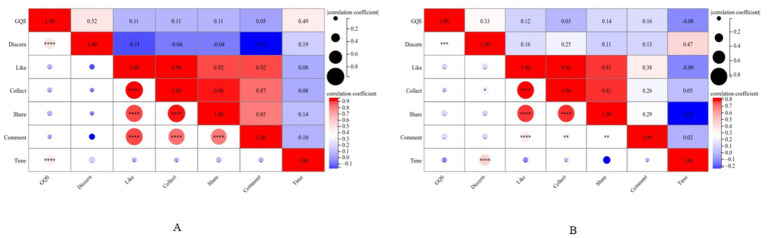
Spearman correlation analysis between different video variables. **(A)** Spearman correlation analysis of the TikTok video variables. **(B)** Spearman correlation analysis between BiliBili video variables.

We used Spearman correlation analysis to analyze the relationships between the basic information of videos on TikTok and BiliBili and the GQS and modified DISCERN ratings. There is a strong positive correlation between video likes, collects, shares, and comments on both platforms. In addition, a fascinating phenomenon can be observed in the videos of both platforms; the GQS score on the TikTok platform and the modified Discern score on the BiliBili platform seem to have strong positive correlations with video length. In addition, on both platforms, there is not a strong correlation between GQS and modified concern ratings or between GQS and liking, collecting, sharing and commenting (Figure 5). In addition, we found that the longer the video duration is, the worse the video, such as, collect, share, and comment data, which suggests that overly long videos may not be able to capture the viewer’s attention.

The higher DISCERN scores on BiliBili reflect superior information reliability characteristics: videos on this platform more frequently cited evidence sources (68% vs. 31% on TikTok), provided balanced information about treatment benefits and risks (54% vs. 28%), and clearly disclosed content creator credentials (76% vs. 45%). This indicates less misinformation and more trustworthy content rather than merely better production quality. Conversely, TikTok’s marginally higher GQS scores primarily reflected better presentation quality (editing, visual appeal, and information flow) rather than scientific accuracy. Importantly, neither platform’s videos were free from quality concerns: 23% of TikTok videos and 15% of BiliBili videos contained at least one instance of oversimplification or unsupported claims.

#### 3.3.1 Misinformation and low-quality content analysis

We performed detailed analysis of videos scoring ≤2 on both GQS and DISCERN scales (*n* = 27, 13.5% of total sample). Clear instances of misinformation were identified in 18 videos (9% of total; TikTok: *n* = 13, BiliBili: *n* = 5), including:

1. Exaggerated efficacy claims (*n* = 7): Videos stating radiotherapy “completely eliminates all cancer cells without side effects” or guaranteeing cure rates without acknowledging disease-specific variation.
2. Omission of critical safety information (*n* = 6): Failure to mention contraindications, serious side effects, or need for medical supervision.
3. Promotion of unproven alternatives (*n* = 3): Suggesting herbal remedies or dietary supplements could replace or significantly enhance radiotherapy effectiveness without scientific evidence.
4. Technical inaccuracies (*n* = 2): Fundamental errors about radiation physics or treatment mechanisms.

Low-quality videos were significantly shorter (median duration: 67 s vs. 645 s for high-quality videos, *p* < 0.001) and more likely to originate from non-professional sources (67% from laypeople/commercial accounts vs. 8% for high-quality videos, *p* < 0.001). Notably, engagement metrics did not predict quality—some misinformation-containing videos had high like/share counts, highlighting the inadequacy of popularity as a quality indicator.

## 4 Discussion

This cross-sectional study is the first to systematically assess the quality and reliability of radiotherapy science videos on two major Chinese short video platforms (BiliBili and TikTok). The results showed that the overall quality (median GQS score of 3) and reliability (median DISCERN score of 3) of the videos on both platforms were at an intermediate level but that the DISCERN score of BiliBili was significantly higher than that of TikTok (*p* < 0.05), suggesting that the reliability of the information was superior, whereas the GQS score of TikTok was slightly higher, suggesting that the platform radiotherapy-related science videos may be of better quality. The source of the video had a remarkable impact on quality: videos produced by radiation therapy specialists (71% in BiliBili and 88% in TikTok) were more scientifically sound in terms of the principles of radiotherapy techniques, descriptions of indications, and management of side effects. In addition, TikTok videos had considerably higher interaction metrics (median number of likes and shares of 353 and 81, respectively) than BiliBili videos did (7.5 and 5.5), but their videos were shorter in length (median 98 s) and limited in depth of content, whereas BiliBili videos were much longer (median 1,391.5 s), which is more conducive to systematic knowledge transfer in radiotherapy. Our data analysis revealed that the vast majority of short videos on the popular science of radiation therapy on the two major short-video platforms failed to systematically present the complete knowledge system of radiation therapy. More alarmingly, oversimplified presentations with insufficient citations of clinical evidence were prevalent in both platforms, and this type of information bias may pose a potential risk of misleading patients in their treatment choices and health management decisions.

### Platform characteristics and their implications for health communication

BiliBili and TikTok represent distinct paradigms in short video content delivery, with important implications for medical information dissemination. TikTok (known as Douyin in China) is characterized by: (1) extremely short video format (typically 15–60 s, maximum 10 min), (2) algorithm-driven content discovery prioritizing viral potential and engagement metrics, (3) younger user demographic (predominantly 16–35 years), and (4) emphasis on entertainment and trend-following. BiliBili, originally an anime and gaming platform that evolved into comprehensive video-sharing service, features: (1) flexible video length with no strict time limits, supporting long-form educational content, (2) community-driven content discovery with emphasis on niche interest groups, (3) unique “bullet comment” (danmaku) feature allowing real-time overlay commentary, and (4) relatively older, education-oriented user base. These structural differences fundamentally shape how medical information can be conveyed: TikTok’s format inherently limits depth and nuance, favoring emotional impact and memorability, while BiliBili accommodates systematic knowledge transmission but requires higher viewer commitment. Our findings reflect these platform affordances—BiliBili’s reliability advantage stems from its accommodation of comprehensive explanations, while TikTok’s engagement success reflects optimization for viral, simplified content.

Many studies have assessed the quality and reliability of short videos as a source of information on malignant tumors, such as those of colorectal cancer (16), laryngeal cancer (17), gastric cancer (18), and skin cancer (19), but no cross-sectional studies have evaluated the quality and reliability of radiation therapy, which is one of the essential oncological treatments. Radiotherapy’s dependence on multidisciplinary collaboration and technical precision—from target delineation to dosimetric optimization—requires that educational content maintains strict scientific standards (20–22). However, short video platforms present a structural tension: their fragmented, entertainment-oriented format conflicts with the systematic knowledge transfer needed for complex medical topics. Content creators must balance accessibility with accuracy, deconstructing technical concepts through visualizations and metaphors while preserving clinical fidelity.

Spearman’s analysis revealed that the video duration of BiliBili was significantly positively correlated with the DISCERN score (*R* = 0.47, *p* < 0.0001), suggesting that longer videos are more conducive to the complete conveyance of complex medical information with greater reliability; the video duration of TikTok was significantly positively correlated with the GQS score (*R* = 0.49, *p* < 0.0001), suggesting that longer science videos on this platform are of greater quality. The interaction indicators (likes, favorites, shares, and comments) on both the TikTok and BiliBili platforms were not significantly associated with the GQS scores. There is no significant correlation between interaction metrics (likes, favorites, shares, and comments) and the length of time spent on both platforms, and sharing is negatively correlated with the length of time spent on the BiliBili platform. There is a strong positive correlation between interaction metrics (likes, comments, shares, and favorites) on both TikTok platforms and a strong positive correlation between interaction metrics (likes, shares, and favorites) on the BiliBili platform, suggesting that positive user feedback tends to exhibit multiple forms of interaction, which is consistent with previous reports (23).

In the era of digital health communication, short video platforms have become a core channel for the public to acquire medical knowledge (9, 24, 25), including radiation therapy. This study systematically reveals for the first time the current quality and dissemination characteristics of radiotherapy videos on two mainstream platforms (BiliBili and TikTok) in China, providing empirical evidence for the public to screen scientific and reliable medical information and helping reduce the risk of health decision-making due to information bias. The results suggest that platform characteristics significantly affect the depth of content and dissemination effectiveness: BiliBili’s long video mode is more suitable for systematic knowledge transmission of radiotherapy, whereas TikTok’s fragmented dissemination needs to be alerted to the risk of knowledge fragmentation. For medical practitioners, this study reveals the structural contradiction between professional content and user needs: videos produced by radiotherapy experts are scientifically superior but not interactive enough, and there is an imminent need to enhance the attractiveness of dissemination through technological innovations (e.g., dynamic dose distribution visualization, interactive radiotherapy process simulation). In addition, the study calls for platforms to optimize the algorithmic recommendation mechanism, incorporate content quality scores (e.g., GQS/DISCERN) into the weighting system, and prioritize the display of authoritative information to construct an ecology of health information on the basis of evidence-based medicine. These findings provide a crucial decision-making basis for public health departments to formulate online health communication norms and for medical organizations to carry out accurate scientific practices.

The core strength of this study lies in the first multidimensional quality assessment of radiotherapy popularization content on two mainstream short video platforms in China. The study adopts the dual assessment system of GQS and DISCERN, taking into account the quantitative analysis of information quality and clinical decision support efficacy; by comparing the platform differences (e.g., the in-depth analysis of BiliBili’s long videos versus the efficient dissemination of TikTok’s short videos), the study reveals the dynamic correlation between the content production mode and dissemination effect; and by combining the multimodal dissemination theories, the study provides methodological references for subsequent studies.

The study has the following limitations: first, the sample only includes videos ranked in the top 100 of the search rankings of each platform, which may have algorithmic bias; second, the evaluation tool is designed mainly for textual content, and there is a lack of quantitative indices for the audio–visual elements, such as 3D modeling and animation demonstrations, which are unique to short videos, which may underestimate the cognitive synergism of multimedia technology; third, the data collection is limited to Chinese platforms, and extrapolation of the conclusions to other languages; third, the data collection is limited to the Chinese platform, so it is prudent in extrapolating the conclusions to other languages and cultural backgrounds; fourth, the video updating dynamics and long-term dissemination effects are not tracked. In the future, we need to develop cross-modal assessment tools, expand the sample coverage, and continue to analyze users’ cognitive behaviors in depth.

## 5 Conclusion

In this study, a systematic evaluation of 200 radiotherapy videos on BiliBili and TikTok reveals that their overall quality and reliability are in the middle range, and there are significant platform differences: BiliBili is better than TikTok in terms of information reliability (DISCERN score), and the latter is more likely to realize user interaction owing to its short and fast dissemination characteristics. However, professional videos generally face the “communication power dilemma.” On the basis of this, a mechanism of “science–communication optimization” is suggested: on the one hand, radiotherapists need to innovate the expression paradigm (e.g., gamified simulation of the radiotherapy process); on the other hand, the platform should establish a quality grading recommendation system to prioritize the videos. On the other hand, the platform should establish a quality grading recommendation system and prioritize the delivery of authoritatively certified content. On the other hand, platforms should establish a quality grading recommendation system and prioritize the delivery of authoritative content. The public needs to strengthen media literacy and prioritize the use of professionally certified video sources when acquiring radiotherapy-related knowledge. This study provides a key chain of evidence for optimizing the ecology of medical science popularization in the digital era.

## Data Availability

The original contributions presented in the study are included in the article/Supplementary material, further inquiries can be directed to the corresponding author.
