# Supplementary material for: Assessing the scientific quality of radiotherapy popularization videos on BiliBili and TikTok: a cross-sectional study
Source: Front Public Health. 2026 Feb 13;14:1668189. doi: 10.3389/fpubh.2026.1668189 (PMC12946092; doi:10.3389/fpubh.2026.1668189)

**Supplementary**

Table S1Global Quality Score (GQS) (Scoring ranges from 1 to 5)

| **GQS Definition** | **Score** |
| --- | --- |
| Poor quality：Specifically, the content is illogical, the mobility is poor, most of the information is missing, and it is useless for patients. | 1 |
| Generally poor quality ：the content logic is poor, although some information is listed, more important information is still missing, and the use of patients is very limited. | 2 |
| Moderate quality：some important information is adequately discussed. | 3 |
| Good quality and flow：Specifically, the video logic is clear and smooth, covering most of the relevant information, which is useful for patients. | 4 |
| Excellent quality and flow：Specifically, the video logic is clear, and the content is very smooth, which is very useful for patients. | 5 |

Table S2 Modified DISCERN quality criteria for assessing the reliability of video. (1 point for answer ‘yes’, 0 point for answer ‘no’)

| Reliability Score |
| --- |
| 1. Is the video clear, concise, and understandable? |
| 2. Are valid sources cited? |
| 3. Is the content presented balanced and unbiased? |
| 4. Are additional sources of content listed for patient reference? |
| 5. Are areas of uncertainty mentioned? |

Table S3 TikTok video source, content, modified Discren score, GQS score, and characteristics of the video

| **Variables** | Like, M (Q₁, Q₃) | Collect, M (Q₁, Q₃) | Share, M (Q₁, Q₃) | Comment, M (Q₁, Q₃) | Time, M (Q₁, Q_3_) | GQS, M (Q₁, Q_3_) | Modified DISCERN M (Q_1_, Q_3_) |
| --- | --- | --- | --- | --- | --- | --- | --- |
| **Video contents (n = 100)** |  |  |  |  |  |  |  |
| **Cases reports (n = 6)** | 85.00 (39.00, 138.50) | 20.50 (8.75， 29.25) | 8.00 (3.25，20.25) | 9.00 (4.5，15.75) | 104.50 (86.50，208.75) | 2.00 (2.00, 2.00) | 1.00 (1.00，1.75) |
| **Introduction to Radiotherapy Methods (n = 27)** | 440.00 (110.00, 2079.00) | 135.00 (39.50, 962.50) | 108.50 (17.25, 492.00) | 51.00 (13.00, 182.00) | 87.00 (41.00, 135.00) | 3.00 (2.00, 3.00) | 2.00 (2.00, 2.50) |
| **Adverse reaction (n = 16)** | 454.00 (262.75, 1045.00) | 186.50 (98.75, 327.75) | 98.50 (65.25, 316.75) | 34.50 (19.25, 60.25) | 93.50 (60.00, 132.00) | 3.00 (3.00, 4.00) | 2.00 (2.00, 2.00) |
| **Radiotherapy cost (n = 3)** | 1890.00 (996.50, 2549.00) | 1683.00 (853.00, 1933.00) | 1058.00 (535.50, 1173.00) | 97.00 (50.50, 208.00) | 75.00 (47.50, 129.00) | 2.00 (2.00, 2.00) | 2.00 (1.50, 2.00) |
| **Process Introduction (n = 8)** | 513.50 (160.50, 838.75) | 172.50 (48.50, 505.75) | 272.50 (90.75, 421.25) | 45.50 (14.50, 104.25) | 96.50 (71.50, 128.25) | 2.50 (2.00, 3.00) | 2.00 (2.00, 2.25) |
| **Notes for Attention (n = 6)** | 324.00 (237.25, 404.00) | 116.00 (32.50, 298.50) | 47.50 (19.25, 80.25) | 18.50 (17.25, 20.50) | 99.00 (67.75, 122.75) | 3.00 (3.00, 3.75) | 2.00 (2.00, 2.00) |
| **Education and Popularization (n = 34)** | 309.00 (106.25, 725.25) | 65.50 (15.75, 230.00) | 44.50 (7.00, 180.00) | 31.00 (7.25, 84.25) | 101.00 (78.50, 146.00) | 3.00 (3.00, 4.00) | 2.00 (2.00, 2.00) |
| **Video source (n = 100)** |  |  |  |  |  |  |  |
| **News agencies (n = 1)** | 1584.00 (1584.00, 1584.00) | 1054.00 (1054.00, 1054.00) | 2054.00 (2054.00, 2054.00) | 14.00 (14.00, 14.00) | 66.00 (66.00, 66.00) | 3.00 (3.00, 3.00) | 2.00 (2.00, 2.00) |
| **Individual science communicators (n = 2)** | 224.50 (164.25, 284.75) | 60.00 (43.50, 76.50) | 46.00 (34.50, 57.50) | 68.00 (42.50, 93.50) | 326.00 (282.00, 370.00) | 2.00 (2.00, 2.00) | 1.00 (1.00, 1.00) |
| **Other medical specialist (n = 5)** | 124.00 (105.00, 289.00) | 23.00 (18.00, 59.00) | 15.00 (8.00, 40.00) | 16.00 (13.00, 46.00) | 106.00 (77.00, 137.00) | 3.00 (2.00, 4.00) | 2.00 (2.00, 2.00) |
| **Medical institution (n = 4)** | 13.50 (7.50, 816.25) | 3.00 (1.50, 548.75) | 3.00 (1.50, 267.50) | 3.50 (0.75, 84.25) | 110.00 (66.50, 144.25) | 2.50 (1.75, 3.25) | 1.50 (1.00, 2.25) |
| **Radiotherapy specialist (n = 88)** | 391.50 (135.00, 937.25) | 129.50 (34.50, 502.75) | 93.50 (13.75, 340.75) | 32.00 (12.00, 91.75) | 98.00 (61.00, 130.25) | 3.00 (2.00, 3.00) | 2.00 (2.00, 2.00) |

Table S4 BiliBili video source, content, modified Discren score, GQS score, and characteristics of the video

| **Variables** | Like, M (Q₁, Q₃) | Collect, M (Q₁, Q₃) | Share, M (Q₁, Q₃) | Comment, M (Q₁, Q₃) | Time, M (Q₁, Q₃) | GQS, M (Q₁, Q₃) | Modified DISCERN M (Q₁, Q_3_) |
| --- | --- | --- | --- | --- | --- | --- | --- |
| **Video contents (n = 100)** |  |  |  |  |  |  |  |
| **Introduction to Radiotherapy Methods (n = 9)** | 18.00 (15.00, 22.00) | 59.00 (35.00, 77.00) | 16.00 (14.00, 32.00) | 0.00 (0.00, 1.00) | 319.00 (218.00, 2220.00) | 3.00 (2.00, 3.00) | 3.00 (3.00, 4.00) |
| **Adverse reaction (n = 1)** | 8.00 (8.00, 8.00) | 2.00 (2.00, 2.00) | 5.00 (5.00, 5.00) | 0.00 (0.00, 0.00) | 399.00 (399.00, 399.00) | 4.00 (4.00, 4.00) | 4.00 (4.00, 4.00) |
| **Process Introduction (n = 11)** | 16.00 (11.00, 108.50) | 54.00 (14.50, 276.50) | 18.00 (5.50, 231.50) | 1.00 (0.00, 3.00) | 496.00 (294.00, 1326.50) | 3.00 (2.00, 3.50) | 4.00 (3.00, 4.00) |
| **Radiation therapy protection (n = 3)** | 1.00 (1.00, 20.50) | 11.00 (7.00, 47.00) | 7.00 (4.50, 22.50) | 0.00 (0.00, 1.50) | 3293.00 (1716.50, 3769.00) | 1.00 (1.00, 1.00) | 2.00 (1.50, 2.50) |
| **Introduction of radiotherapy equipment and drugs (n = 5)** | 10.00 (2.00, 11.00) | 25.00 (10.00, 26.00) | 5.00 (4.00, 12.00) | 1.00 (0.00, 4.00) | 2515.00 (2014.00, 3556.00) | 2.00 (2.00, 3.00) | 3.00 (3.00, 3.00) |
| **Education and Popularization (n = 71)** | 4.00 (2.00, 14.50) | 11.00 (3.50, 48.50) | 3.00 (0.00, 12.00) | 0.00 (0.00, 1.00) | 1440.00 (774.00, 2639.50) | 2.00 (2.00, 3.00) | 4.00 (3.00, 4.00) |
| **Video source (n = 100)** |  |  |  |  |  |  |  |
| **Individual science communicators (n = 17)** | 12.00 (6.00, 24.00) | 25.00 (8.00, 79.00) | 19.00 (9.00, 60.00) | 0.00 (0.00, 2.00) | 216.00 (171.00, 325.00) | 3.00 (3.00, 3.00) | 3.00 (3.00, 3.00) |
| **Other medical specialist (n = 1)** | 3.00 (3.00, 3.00) | 2.00 (2.00, 2.00) | 5.00 (5.00, 5.00) | 0.00 (0.00, 0.00) | 2637.00 (2637.00, 2637.00) | 3.00 (3.00, 3.00) | 3.00 (3.00, 3.00) |
| **Medical institution (n = 11)** | 18.00 (11.00, 45.00) | 53.00 (13.50, 98.00) | 13.00 (5.50, 60.00) | 1.00 (0.00, 3.50) | 399.00 (108.00, 834.00) | 2.00 (2.00, 4.00) | 4.00 (3.00, 4.00) |
| **Radiotherapy specialist (n = 71)** | 4.00 (1.50, 15.00) | 12.00 (4.00, 50.00) | 3.00 (0.00, 13.50) | 0.00 (0.00, 1.00) | 1890.00 (1183.00, 2935.00) | 2.00 (2.00, 3.00) | 4.00 (3.00, 4.00) |

Supplementary Fig. 1. likes (A), collects (B), shares (C), and comments (D) based on videos on TikTok and BiliBili.


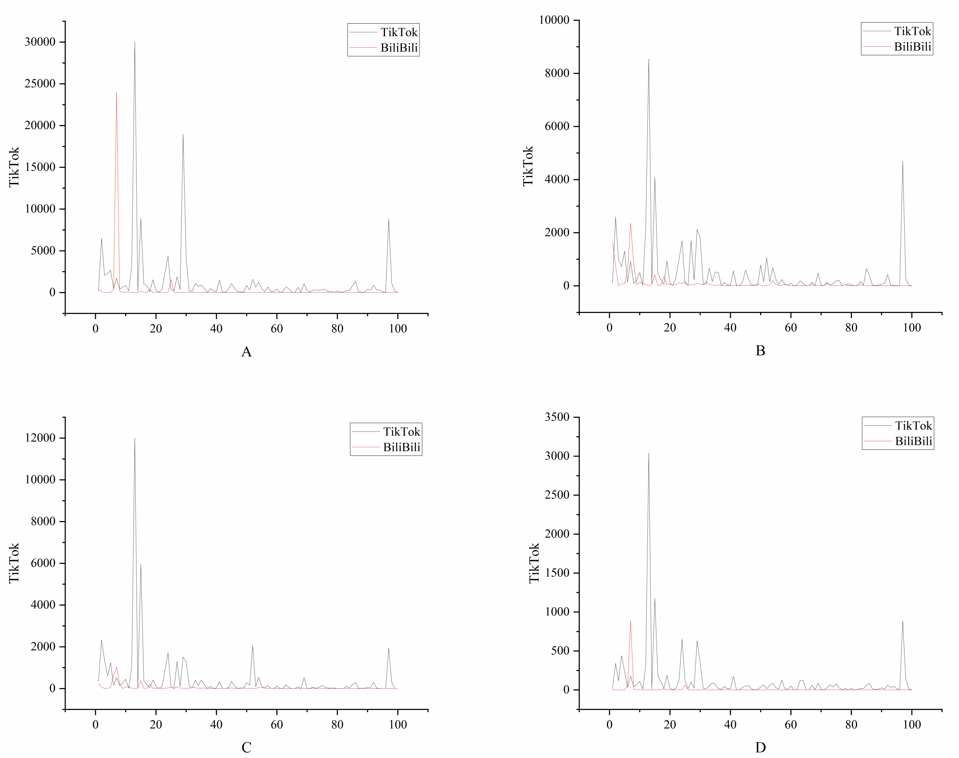

Supplement: Supplementary file 1 [file Data_Sheet_1.docx]
